# AfSwi6 Regulates the Stress Response, Chlamydospore Production, and Pathogenicity in the Nematode-Trapping Fungus *Arthrobotrys flagrans*

**DOI:** 10.3390/microorganisms12091765

**Published:** 2024-08-26

**Authors:** Shao-Xiang Linghu, Yu Zhang, Jia-Fang Zuo, Ming-He Mo, Guo-Hong Li

**Affiliations:** State Key Laboratory for Conservation and Utilization of Bio-Resources in Yunnan, School of Life Sciences, Yunnan University, Kunming 650091, China

**Keywords:** *Arthrobotrys flagrans*, *AfSwi6*, trap, chlamydospores, pathogenicity, transcriptome

## Abstract

Nematode-trapping (NT) fungi are a major resource for controlling parasitic nematodes. *Arthrobotrys flagrans*, as a typical NT fungus, can capture nematodes by producing three-dimensional nets. The APSES transcription factor *Swi6* plays a vital role in fungal growth and the pathogenicity of pathogens. In this study, we characterized *AfSwi6* via gene disruption using the homologous recombinant method and transcriptome sequencing. Knockout of the *AfSwi6* gene caused defects in mycelial growth, trap formation and pathogenicity, chlamydospore production, and stress response. Moreover, the transcriptome data indicated that *AfSwi6* was related to DNA repair, stress response, and plasma membrane fusion. The result showed that *AfSwi6* has a significant effect on trap development and chlamydospore production in *A. flagrans*.

## 1. Introduction

Nematode-trapping (NT) fungi capture prey by forming traps [1]. In NT fungi, low-nitrogen conditions lead to a lifestyle change from saprophytic to predacious, and trap development is a crucial indicator of this [2]. *Arthrobotrys flagrans* [*Duddingtonia flagrans*, (Duddington) R. C. Cooke] can capture and kill nematodes by producing adhesive nets. *A. flagrans* has been used for biocontrol of parasitic nematodes, especially those that are parasitic upon animals. Biocontrol agents created with this species have now been utilized successfully in a variety of animals, including buffalo, sheep, pigs, and horses [3,4,5,6]. In addition, it has been reported that *A. flagrans* may be used for control of plant pathogenic nematodes such as *Meloidogyne incognita* (Kofoid & White) and *Xiphinema index* [7,8]. Compared with other NT fungi, its ability to produce chlamydospores gives *A. flagrans* greater potential for biological control.

The APSES (Asm1p, Phd1p, Sok2p, Efg1p, and StuAp) protein family comprises transcription factors that are part of the basic helix–loop–helix (bHLH) class [9], which play significant roles in the sprouting of conidia, the growth of hyphae, cell differentiation, and secondary metabolism [10]. SWI6, a member of this family, has been proven to be a target of SLT2/Mpk1 in fungi [11,12]. The *Swi6* gene has long been recognized as one of the genes that has an effect in the mating-type transformation of yeast. It is necessary for two heterogeneous transcription factor complexes, SBF and MBF, in the G1/S phase of yeast [13]. The deletion of *Scswi6* in *Saccharomyces cerevisiae* affected the frequency of meiotic recombination, oxidative stress, DNA repair, and cell wall biosynthesis [14]. *MoSwi6* regulates extracellular enzyme production and activity, as well as pathogenicity, in *Magnaporthe oryzae* [15]. In *Fusarium graminearum, FgSwi6* can affect fungal growth, drug resistance, metabolite production, and toxicity [16]. The deletion of *CgSWI6* impaired tolerance to acanthomycin in *Candida glabrata* [17]. According to a study on *Ganoderma lucidum*, the sensitivity of cell walls to pressure is affected by *GlSwi6*, which also regulates the growth of the mycelium and the synthesis of secondary metabolites [18]. In *Metarhizium rileyi, MrSwi6* had a meaningful impact on the formation of dimorphic transitions, conidia, and microbial nuclei and also impacted virulence [19]. In *Ceratocystis fimbriata*, fungal growth, cell wall integrity (CWI), and pathogenicity are all dependent upon *CfSwi6* [20]. In *Beauveria bassiana,* the growth of the mycelium, the formation of asexual spores, and virulence are significantly impacted by *BbSwi6* [21]. Finally, *AoSwi6* has been found to be crucial in mycelial growth and conidia formation and also to impact responses to environmental stress, pathogenicity, and metabolism in *Arthrobotrys oligospora* [22]. 

In the present study, we investigated the impact of *AfSwi6* on *A. flagrans* by analyzing phenotypic alterations caused by gene disruption. We found that *AfSwi6* is vital for mycelium growth, chlamydospore formation, and pathogenicity in *A. flagrans*. In addition, the transcriptome data revealed that *AfSwi6* was involved in DNA damage repair, stress response, and plasma membrane fusion. 

## 2. Materials and Methods

### 2.1. Strains and Culture

*Arthrobotrys flagrans* YMF1.07536 was conserved in the Microbial Library of the Germplasm Bank of Wild Species of Southwest China. It was cultured on PDA medium (Table S1), the mutant strains were cultured on PDA medium containing hygromycin B (100 μg/mL), and the complementation of the *AfSwi6* gene strains (*AfSwi6^C^*) were cultured on PDA medium containing 50 μg/mL of nourseothricin [23]. *Caenorhabditis elegans* was inoculated into NGM medium (Table S1) containing the nematode food, *Escherichia coli* (OP50), and incubated at 20 °C in a thermostatic biochemical incubator (Shanghai Heng Scientific Instrument Co., Shanghai, China).

### 2.2. Sequence and Phylogenetic Analysis

**Figure 1:**
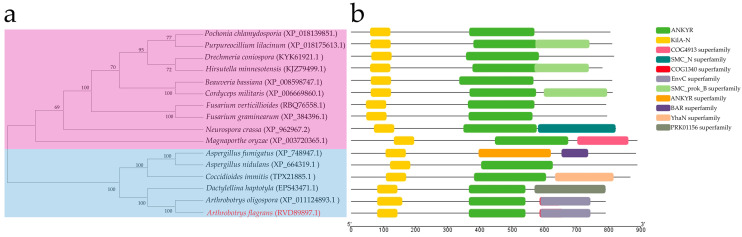
Conserved domain and phylogenetic analyses of SWI6. (**a**) Neighbor-joining tree based on 1000 repeated bootstrap values. GenBank accession numbers are provided in brackets. (**b**) Conserved domain analysis of SWI6.

From the genome data of *A. flagrans* CBS 565.50, the DNA sequence of *AfSwi6* was obtained [23]. The software package MEGA-X (http://www.megasoftware.net/, accessed on 1 March 2024) was employed for phylogenetic analysis using the neighbor-joining (NJ) method with a bootstrap test set up 1000 times. The results were displayed in a phylogenetic tree (Figure 1a). The NCBI online tool Conserved Domain Search Service (https://www.ncbi.nlm.nih.gov/Structure/cdd/wrpsb.cgi, accessed on 1 March 2024) was also used, and protein-conserved domains were predicted (Figure 1b) and analyzed using TBtools software (Version number: 2.056) [24].

### 2.3. Plasmid Construction and Protoplast Transformation

Knockout of the *AfSwi6* gene was performed by homologous recombination using the primer groups Ko0885-up-for/rev, Ko0885-down-for/rev, and Ko0885-hyg-for/rev (Table S2), with the PCR amplification corresponding to fragments of 1124 and 1087 bp on the flanks of the *AfSwi6* gene and the 2121 bp *hph* gene. Meanwhile, the framework complementing the *AfSwi6* gene is composed of a 2000 bp upstream promoter and a downstream terminator of about 1000 bp, as well as nourseothricin as a resistance screening marker. The specific vector construction methods are described in the Materials and Methods section of the Supplementary Materials.

The mycelium of *A. flagrans* was collected with STC buffer (Table S1), and the protoplasts were prepared with snailase (2%, Solarbio) and cellulose (2%, Solarbio). The knockout fragments and the framework complementing the *AfSwi6* gene were then transformed using PTC buffer (Table S1). Finally, the transformants were screened with medium containing hygromycin B (100 μg/mL) [23] and nourseothricin (50 μg/mL), respectively.

### 2.4. Hyphae Growth and Analysis of Resistance 

The WT and Δ*AfSwi6* strains were cultured on PDA and TYGA media (Table S1) at 28 °C for 5 days. The experimental details and calculation of the relative growth inhibition (RGI) [22] are given in the Materials and Methods section of the Supplementary Materials.

### 2.5. Hyphae Morphology, Lipid Droplets, and Glycogen Staining 

The WT and Δ*AfSwi6* strains were cultured on PDA for 4–5 days. The mycelium was stained with 10 μg/mL of Calcofluor white (CFW, Sigma, Shanghai, China) to observe the length of the cells and the deletion of the diaphragm. For glycogen detection, we stained the samples with Lugol’s iodine solution (Sigma, Shanghai, China) for 1 min. Lipid droplets were stained with BODIPY (10 μg/mL, Sigma, 790389, Shanghai, China) for half an hour, after which the excess dye was washed away with phosphate buffer solution (PBS) [23].

### 2.6. Trap Formation and Assays of Pathogenicity 

The WT and Δ*AfSwi6* strains were inoculated onto WA medium (Table S1), respectively, at 28 °C for 3 days. Then, approximately 300 *C. elegans* were added to the Petri dishes. The number of traps and captured nematodes was observed for 48 h. At the same time, a cryo-scanning electron microscope (Cryo-SEM) was used to observe the shapes of the traps in the WT and Δ*AfSwi6* strains. In addition, any changes inside the cells of the chlamydospores, hyphae, and traps were observed using a transmission electron microscope (TEM). For TEM observation, samples were fixed with 2.5% glutaraldehyde and kept at 4 °C for at least 12 h. For extracellular protease activity, the WT and Δ*AfSwi6* strains were cultured using LMZ medium (Table S1) at 28 °C and 180 rpm for 7 days. Fermentation broth was added to the wells (0.6 cm in diameter) in the dish with 10% skimmed milk agar medium; after culturing for 24 h, the diameter of the protein hydrolysis circle was measured.

### 2.7. Transcriptome Sequencing 

The experiments in this section include sample preparation, detailed sampling procedures, RNA extraction methods, library pre-processing, reading techniques, and software methods for processing the data, and the experimental details are described in the Materials and Methods section of the Supplementary Materials.

### 2.8. Real-Time Fluorescent Quantitative PCR Assay

Real-time fluorescent quantitative PCR was performed using AceQ qPCR SYBR Green Master Mix (Vazyme, Nanjing, China) on a Roche LightCycler 480 system (Roche Applied Science, Penzberg, Germany) with the gene-specific primer pairs (Table S2). The amplification conditions were 94 °C for 5 s and 60 °C for 30 s for 40 cycles. The glyceraldehyde-3-phosphate dehydrogenase (*AfGpd*) gene was used as an internal control, and the 2^−∆∆CT^ method was used to calculate the relative transcription level. All the assays were repeated at least three times.

### 2.9. Statistical Analyses

In this study, statistical analyses were performed using GraphPad Prism v8.3. All the experiments were repeated at least three times, as well as using more than three replicates for each treatment. We used the Holm–Sidak test to determine significant differences between the mutants and the WT. Data were expressed as means ± standard deviations; *p* < 0.05 indicated a significant difference from the WT strain.

## 3. Results

### 3.1. Analysis of the Phylogenetic Tree and Protein Conserved Domains and the Deletion of AfSwi6

Through comparison with the amino acid sequence of *S. cerevisiae* SWI6 (NP_013283.1), the homologous protein AfSWI6 (RVD89897.1) in *A. flagrans* was obtained. Through comparison with BLAST, the orthologs of SWI6 in the filamentous fungi were downloaded. The orthologs of SWI6 in the NT fungi form an independent evolutionary branch (Figure 1a), with AfSwi6 having the greatest similarity to AoSwi6. The coding sequences (CDSs) for the *AfSwi6* gene contain 2346 bp that code 781 amino acids and include four protein-conserved domains (Figure 1b), these being KILA, KANKYR, COG1340, and the EnvC superfamily. 

The gene *AfSwi6* was disrupted using the homologous recombination method (Figure S1a). PDA medium containing hygromycin B was used to screen the positive strains. Verification was obtained via PCR with the primers ko0885-up-for and ko0885-down-rev, ko0885-hyg-for and ko0885-hyg-rev, and ko0885-gene-for and ko0885-gene-rev (Figure S1a,b and Table S2). Ultimately, three knockout strains, Δ*AfSwi6-1*, Δ*AfSwi6-3,* and Δ*AfSwi6-6,* were successfully obtained (Figure S1b). The expression level of the *AfSwi6* gene in the mutant strain was zero (Table S3). This was also verified by qPCR (Figure S2a,b). Meanwhile, three complementary transformants of the *AfSwi6* gene (*AfSwi6^C^*) were obtained in the study (Figure S3a,b). 

### 3.2. AfSwi6 Affects Fungal Growth and Resistance to Stress 

**Figure 2:**
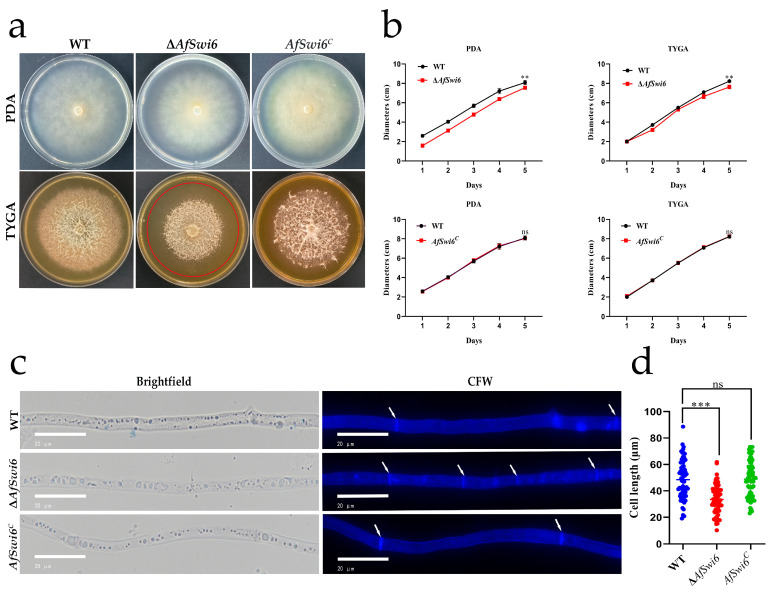
The growth of WT and Δ*AfSwi6* and *AfSwi6^C^* strains. (**a**) Colony morphology of WT, Δ*AfSwi6,* and *AfSwi6^C^* cultivated on two media, and the red circles indicate colony size. (**b**) Colony diameters of WT, Δ*AfSwi6,* and *AfSwi6^C^* cultivated on two media. (**c**) WT, Δ*AfSwi6,* and *AfSwi6^C^* strains were stained with 10 μg/mL of CFW. (**d**) Measuring mycelial cell length (n = 68 samples, average ± SD) (**, *p* < 0.01; ***, *p* < 0.001; ns, no significant).

Among the Δ*AfSwi6* mutants, there was less growth than that in the WT strains cultured on the PDA and TYGA media at 28 °C for 5 days (Figure 2a). The colony diameters were lower in the Δ*AfSwi6* strains than in the WT strains (Figure 2b). Compared with the WT, the Δ*AfSwi6* strains had more hyphal septa and shorter hyphal cells (Figure 2c). Data for the lengths of 68 hyphae cells in a random field showed that the hyphae cells became shorter in the mutants (Figure 2d). The growth and hyphal septa of the *AfSwi6^C^* strain were consistent with those of the WT (Figure 2a–d).

**Figure 3:**
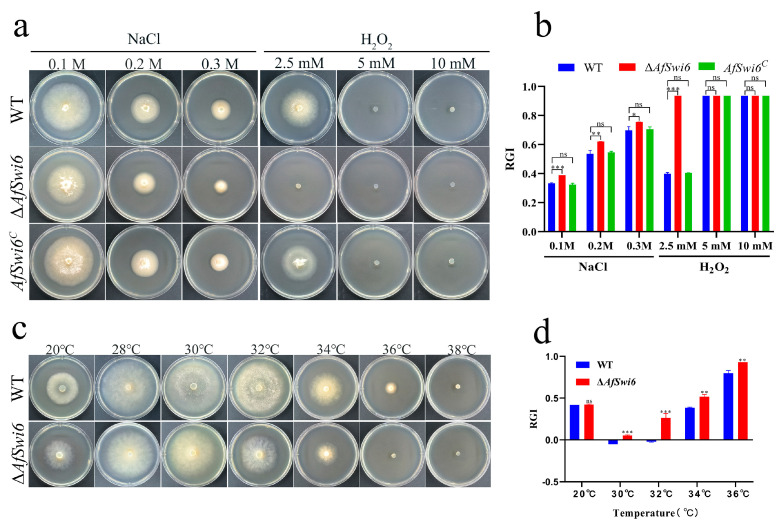
The growth of the WT, Δ*AfSwi6,* and *AfSwi6^C^* on PDA medium containing different stress agents. (**a**) The growth of the WT, Δ*AfSwi6,* and *AfSwi6^C^* on PDA containing NaCl and H_2_O_2._ (**b**) The RGIs of the WT, Δ*AfSwi6,* and *AfSwi6^C^* on PDA containing NaCl and H_2_O_2_. (**c**) The growth of the WT and Δ*AfSwi6* on PDA at different temperatures. (**d**) The RGIs of the WT and mutants at different temperatures. (*, *p* < 0.05; **, *p* < 0.01; ***, *p* < 0.001; ns, no significant)

The WT and Δ*AfSwi6* mutants were cultured at 28 °C for 5 days on PDA media containing different stress reagents, and the RGIs were calculated. The results showed that compared with the WT, for three gradient concentrations of the osmotic reagent NaCl (0.1, 0.2, and 0.3 M), the decreases in the colony diameter and RGIs in Δ*AfSwi6* were 5.64%, 8.47%, and 5.61% greater, respectively, than those in the WT (Figure 3a,b). The mutants showed high sensitivity when exposed to the oxidative reagent H_2_O_2_ at a concentration of 2.5 mM, with the RGI being 53.54% higher than that in the WT (Figure 3b). In addition, when the H_2_O_2_ concentration increased to 5 mM, the WT and mutants were no longer able to grow (Figure 3a). 

Similarly, the mutants exhibited higher sensitivity to sorbitol, SDS, and Congo red than the WT (Figure S4). At a concentration of H_2_O_2_ of 2.5 mM, the phenotype of the *AfSwi6^C^* strain recovered (Figure 3a,b). In addition, the colony morphology of the *AfSwi6^C^* strain in the other experimental groups was consistent with that of the WT (Figure 3a–d and Figure S4). At a 0.1 M concentration of NaCl, the RGI value of 0.32 for the *AfSwi6^C^* strain did not significantly differ from the RGI value of 0.33 for the WT. The other RGI values for the *AfSwi6^C^* strain were also identical to the WT for SDS, Congo red. and Sorbitol. These data suggest that the phenotype of the *AfSwi6^C^* strain was restored after *AfSwi6* gene supplementation. To explore the effects of the *AfSwi6* gene on resilience to heat stress, the WT and Δ*AfSwi6* were cultured at different temperatures for 5 days. Then, taking 28 °C as the control, the colony diameters under various temperatures were measured, and the RGIs were calculated. The result showed that the colony diameters of the WT and mutants decreased when cultured at 20 °C, but the RGIs of the mutants did not change significantly compared with the WT. As the temperature rose to 30 °C and 32 °C, the WT colonies returned to normal growth (Figure 3c). However, the RGIs of the mutants were 10.57% and 28.6% higher than those of the WT at 30 °C and 32 °C, respectively (*p* < 0.001) (Figure 3d). As the temperature rose to 34 °C and 36 °C, the colony diameter of the WT also started to decrease. However, the mutants exhibited a greater RGI change than the WT. Notably, the mutants no longer grew properly at 36 °C, while growth of the WT ceased at 38 °C (Figure 3c). 

### 3.3. AfSwi6 Is Vital for Chlamydospore Formation and the Accumulation of Glycogen and Lipid Droplets

**Figure 4:**
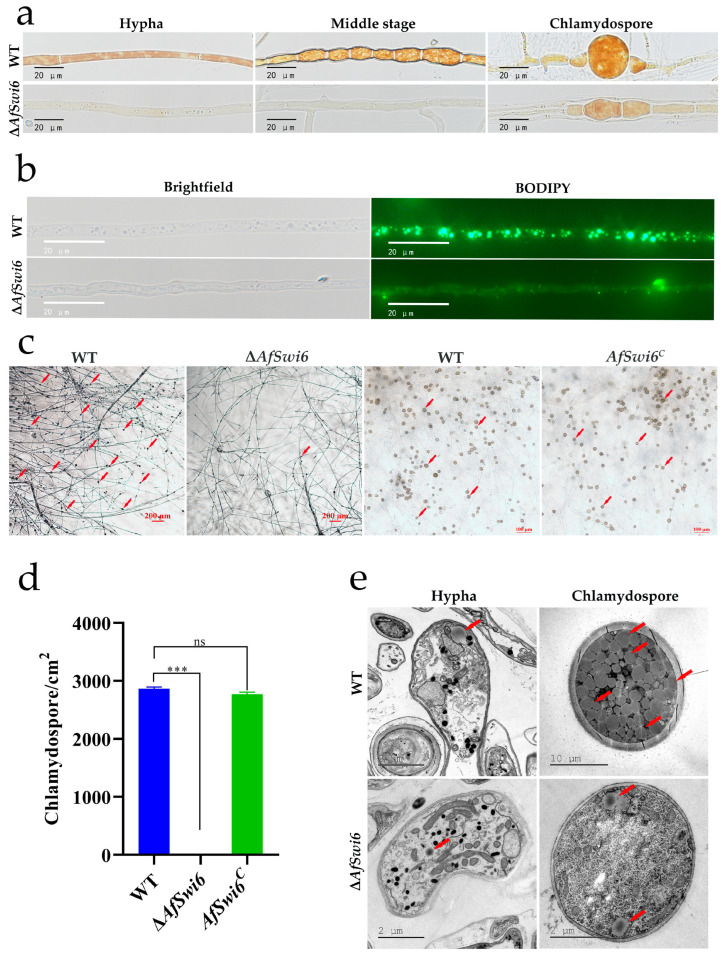
The formation of glycogen, lipid droplets, and chlamydospores was affected by the gene *AfSwi6*. (**a**) Glycogen in different stages of chlamydospore formation in WT and Δ*AfSwi6*. (**b**) Lipid droplets in WT and Δ*AfSwi6*. (**c**) Chlamydospores in WT, Δ*AfSwi6,* and *AfSwi6^C^* cultured on WA medium at 28 °C for 14 days (The red arrow represents chlamydospores). (**d**) Number of chlamydospores in WT, Δ*AfSwi6,* and *AfSwi6^C^* (***, *p* < 0.001; ns, no significant). (**e**) Lipid droplets in WT and Δ*AfSwi6* (The red arrow represents the lipid droplet).

Lugol’s iodine was used to observe the accumulation of glycogen in the different stages of chlamydospore production (Figure 4a). In the WT, glycogen accumulates dynamically, and glycogen is abundant in the chlamydospores. However, the glycogen accumulation in the mutants was lower than that in the WT at all time points. In addition, when the hyphae of the WT began to swell, this indicated the formation of chlamydospores, but there was no significant change in Δ*AfSwi6*. The distribution of lipid droplets in the mycelia of the WT and mutants was then observed through BODIPY staining. Compared with Δ*AfSwi6*, more and larger lipid droplets appeared in the WT (Figure 4b). The number of chlamydospores in the mutants was severely affected after the deletion of the *AfSwi6* gene. On the WA medium, after culturing them at 28 °C for 14 days, only a very small number of chlamydospores were produced in the mutants. The number of chlamydospores in the *AfSwi6^C^* strain did not significantly differ from that in the WT (Figure 4c,d). At the same time, in mature chlamydospores, the number of lipid droplets in the mutants was found to be much lower than that in the WT based on the TEM results (Figure 4e).

### 3.4. AfSwi6 Plays a Key Role in Trap Formation and Pathogenicity in A. flagrans

**Figure 5:**
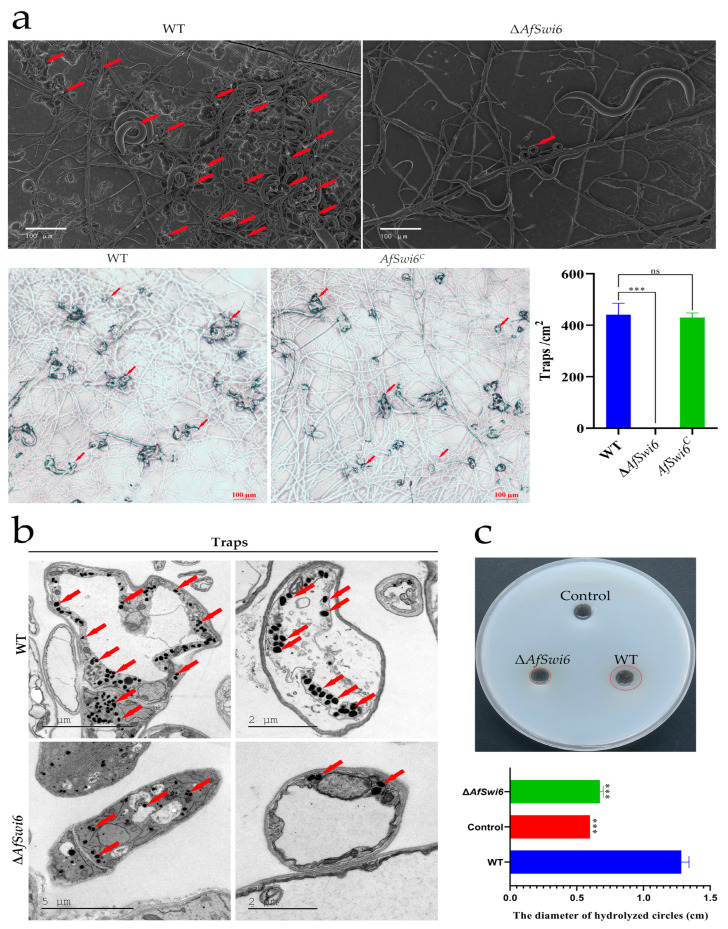
Effects of *AfSwi6* on trap formation and nematode mortality. (**a**) Traps in WT, Δ*AfSwi6,* and *AfSwi6^C^* and number of traps after 36 h of nematode induction (The red arrow represents the traps). (**b**) Electronic densities (EDs) in traps of the WT and mutants (The red arrow represents the electron dense body). (**c**) Determination of extracellular protease activities (The red circle represents the size of the hydrolysis circle) (***, *p* < 0.001; ns, no significant).

*A. flagrans* can capture nematodes by producing traps (adhesive three-dimensional networks). It forms traps only after coming into contact with nematodes. In our previous study, we reported the process of *A. flagrans*’ pathogenicity against nematodes [7]. In this study, *AfSwi6* was found to be involved in trap formation and pathogenicity in *A. flagrans*. In the WT, 24 h after the addition of the nematodes, a large number of traps had formed, and nematodes had already been captured (Figure 5a). Thirty-six hours after the addition of the nematodes, the average number of traps produced by the WT was more than 400/cm^2^, and the nematode capture rate reached 93.33%. In contrast, at the same time point, the mutants had produced very few traps, and the nematode mortality was less than 7%. Further, the number of traps in the *AfSwi6^C^* strain was consistent with that in the WT (Figure 5a). In addition, based on the TEM analysis, fewer electron densities (EDs) were observed in the traps of the mutants compared with the WT (Figure 5b). Furthermore, Δ*AfSwi6* saw a significant decrease in extracellular protease activity (Figure 5c), which is also an indicator of fungal pathogenicity [25].

### 3.5. Transcriptomic Analysis of the WT and ΔAfSwi6

**Figure 6:**
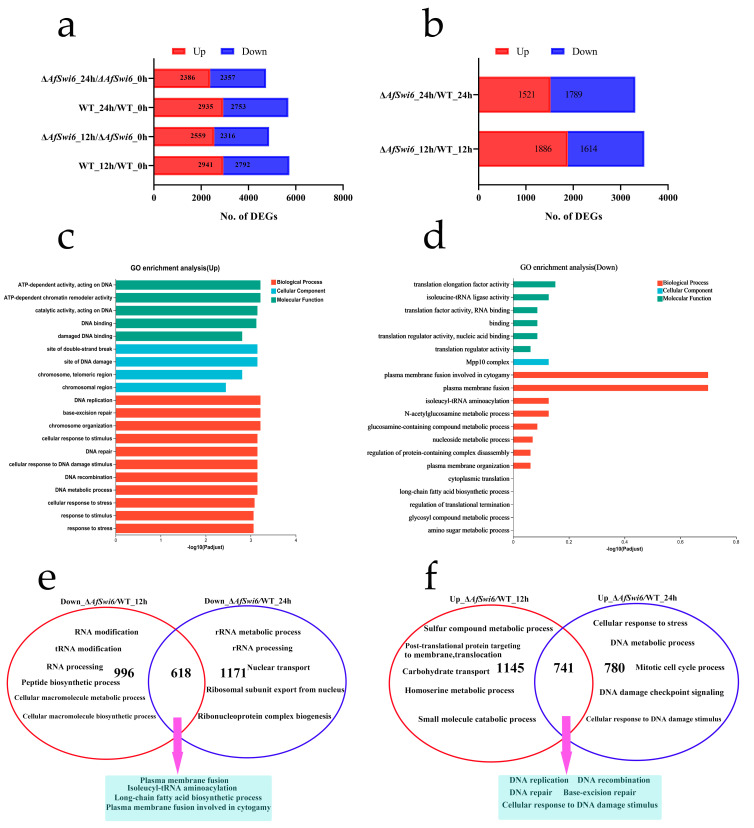
Transcriptomic analysis of differential expression genes (DEGs) in WT and Δ*AfSwi6*. (**a**) The numbers of DEGs in mutants and WT at 12 and 24 h, respectively. (**b**) Differences in the number of DEGs differed in mutants at 12 and 24 h, compared with the WT. (**c**,**d**) GO enrichment analysis of all up- and downregulated DEGs, respectively, in (**b**) (*Padjust* < 0.05). (**e**) Veen analysis of downregulated DEGs in (**b**). (**f**) Veen analysis of upregulated DEGs in (**b**).

To further study *AfSwi6*’s regulatory mechanisms, the transcriptomes of the WT and *ΔAfSwi6* were compared using RNA-seq. The quality control and sequencing data did not reveal any artefacts or contamination, and each group of genes was expressed efficiently (Tables S4 and S5). It is noteworthy that the level of transcription varied at 0 (26.22), 12 (32.56), and 24 h (20.65) after nematode induction. Overall, the number of differential expression genes (DEGs) was higher in the WT than in the mutants (Figure 6a, Table S6). Compared with the WT, 1886 and 1521 DEGs were upregulated in Δ*AfSwi6*, while 1614 and 1789 DEGs were downregulated (Figure 6b, Table S7) at 12 and 24 h, respectively, after nematode induction. Gene Ontology (GO) enrichment analysis showed that the upregulated DEGs were associated with DNA metabolic processes, DNA repair, and cell repair of DNA damage stimuli (Figure 6c, Table S8). However, the downregulated DEGs affected plasma membrane fusion (Figure 6d, Table S9). Among the downregulated DEGs, the first 20 DEGs of the mutant strains were correlated with RNA modification, tRNA modification, tRNA processing, the peptide metabolic process, and the cellular macromolecule biosynthetic process at 12 h (Figure 6e, Table S10). At 24 h, they were mostly associated with the rRNA metabolic process, rRNA processing, nucleocytoplasmic transport, ribosomal subunit export from the nucleus, ribonucleoprotein complex biogenesis, ribosome synthesis, and other biological processes (Figure 6e, Table S11). Among the downregulated genes at the two time points, there were 618 genes that were co-expressed, and GO enrichment analysis indicated that there was a prominent increase in plasma membrane fusion involved in cytogeny, isoleucyl-tRNA aminoacylation, and the biosynthetic process for long-chain fatty acids (Figure 6e, Table S12). However, among the upregulated DEGs in mutants, the first 20 DEGs at 12 h were involved in the metabolic process for sulfur compounds, post-translational protein targeting to the membrane, translocation, the homoserine metabolic process, and carbohydrate transport (Figure 6f, Table S13). At 24 h, the upregulated DEGs were associated with cellular response to DNA damage stimuli, the DNA metabolic process, DNA damage checkpoint signaling, and the mitotic cell cycle process (Figure 6f, Table S14). At the same time, a total of 741 upregulated genes were expressed; these were mainly related to DNA replication and repair and base excision repair (Figure 6f, Table S15).

**Figure 7:**
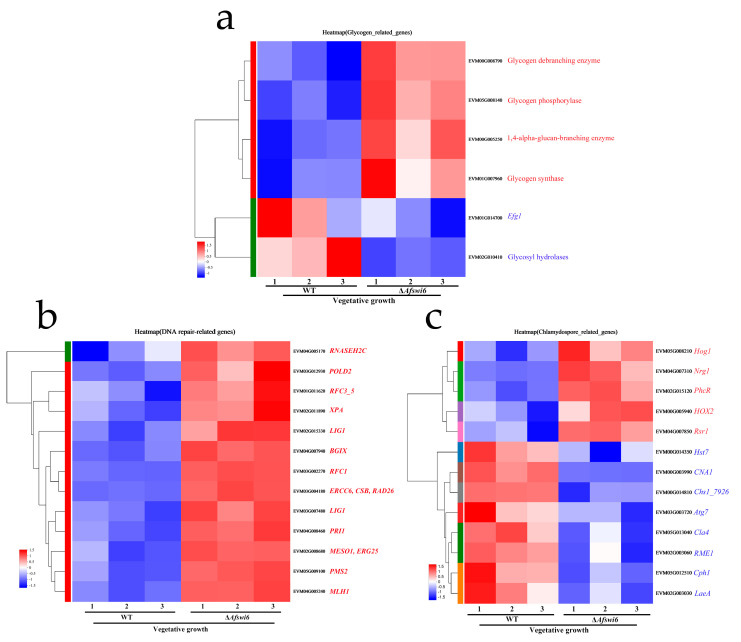
*AfSwi6* affects DNA repair, glycogen synthesis, and chlamydospore formation. (**a**) The expression levels of glycogen-related genes. (**b**) The expression levels of DNA repair-related genes. (**c**) The expression levels of chlamydospore-related genes.

In addition, we analyzed the genes involved in glycogen synthesis, DNA repair, and the regulation of chlamydospores (Figure 7), which were verified by qPCR (Figure S2c). Glycogen degradation requires glycogen phosphorylase and glycogen branching enzyme, both of which are highly expressed in Δ*AfSwi6* (Figure 7a). *Efg1* can regulate the expression of glycogen synthase [26,27], and a lower transcription level was recorded in the Δ*AfSwi6* mutants. The effects of missing *AfSwi6* on the expression patterns of DNA repair-related genes were also investigated. Our findings indicated that the expression of genes involved in DNA repair showed a highly consistent upregulation in the Δ*AfSwi6* mutants (Figure 7b, Figure S2c). Similarly, the genes that regulate the formation of chlamydospores exhibited different expression patterns in the WT and Δ*AfSwi6*. Methyltransferase (*PhcR*), a negative regulator of chlamydospore formation [28], showed high expression in Δ*AfSwi6* (Figure 7c). The genes that regulate the formation of chlamydospores, such as *RME1*, *Atg7*, *can1*, and *Chs1_7926*, all showed lower expression levels (Figure 7c). Notably, in *A. flagrans*, the global regulatory factor *LaeA*, a positive regulator [23], had a key effect on the formation of chlamydospores, showing low expression levels in Δ*AfSwi6* (Figure 7c).

## 4. Discussion

In the present work, disruption of *AfSwi6* was found to slow hyphae growth and shorten the length of the hyphae cells (Figure 2). Further, mycelial growth and the mycelial septa were restored in the *AfSwi6^C^* strain. Such effects have previously been reported to be consistent for various filamentous fungi. *AfSwi6* is critical to the maintenance of cell wall integrity. CWI signaling pathways are vital for regulating the biogenesis and repair of fungal cell walls [29]. *Swi6* involved in the transcriptional regulation of cell wall stress related genes of the CWI-MAPK signal pathway [30]. In *M. oryzae*, the destruction of *MoSwi6* leads to hypersensitivity to oxidative stress and lower activity of extracellular enzymes [15]. In addition, *CfSwi6* is required for CWI in *C. fimbriata* [20]**,** and PKC-SWI6 signals play a key role in CWI and stress response in *A. oligospora* [22]. In the present study, the Δ*AfSwi6* strains showed greater sensitivity (Figure 3) to various stress agents than the WT. However, the sensitivity of the *AfSwi6^C^* strain was consistent with that of the WT (Figure 3a,b and Figure S4). 

Fungi can produce chlamydospores under adverse environmental conditions. Many genes participate in the production of chlamydospores. The formation of chlamydospores in *F. oxysporum* and *Trichoderma virens* is positively regulated by the calcineurin gene *CNA1* and the chitin synthase genes *Chs1_7926* and *Chs1_8917*, respectively [31,32]. In addition, excision of the methyltransferase *phcR* gene in *Leilstonia cerevisiae* has been shown to result in an increase in chlamydospores [28]. It has also been confirmed that knockout of the *UvAtg7*, *UvVEA,* and *UvHOX2* genes causes flaws in chlamydospores [33,34,35] and that deletion of the *AfLaeA* gene fundamentally eliminates the capacity to generate chlamydospores in *A. flagrans* [23], but the number of chlamydospores was restored in the *AfSwi6^C^* strain (Figure 4c,d). In the present study, the accumulation of glycogen and lipid droplets during the formation of the chlamydospores was found to decrease in the mutant stains (Figure 4). The formation of chlamydospores requires the accumulation of glycogen and lipid droplets. In addition, based on the clustering heat map analysis of genes related to glycogen and chlamydospores, we found that the expression of glycogen phosphorylase and glycogen debranching enzyme in Δ*AfSwi6* is relatively high (Figure 7a). Efg1, a transcription factor in the regulation of glycogen synthase expression, had low transcript levels in Δ*AfSwi6* (Figure 7a and Figure S2c). Methyltransferase (*PhcR*), which has a negative regulatory effect on the formation of chlamydospores [28], showed high expression in Δ*AfSwi6* (Figure 7c). However, genes that are positively regulated for chlamydospore formation, such as *AfLaeA, RME1*, *Atg7*, *CNA1*, and *Chs1_7926*, showed lower levels of expression (Figure 7c and Figure S2c). In the present study, the number of chlamydospores significantly decreased, which indicates that *AfSwi6* is essential for chlamydospore formation in *A. flagrans*. 

The deletion of the *AfSwi6* genes also has a severe impact on virulence in *A. flagrans*. In the present study, the deletion of the *AfSwi6* gene reduced the number of traps in Δ*AfSwi6* (Figure 5a). Similarly, the capture rate for nematodes in the mutant strains was less than 7% but reached 93.33% in the WT. The *AfSwi6^C^* strain could produce the same number of traps as the WT (Figure 5a). We also found that the protein hydrolysis activity in the Δ*AfSwi6* strains also decreased compared with that in the WT strains (Figure 5c). These findings demonstrate that the deletion of *AfSwi6* can affect the pathogenicity of *A. flagrans*.

Fungi are important resources for the development of biocontrol agents. But currently, most of the fungal biocontrol agents developed are mycelial or spore-forming agents, and there are some issues such as poor stability, short shelf life, and the need for order production. Chlamydospores have strong stress resistance and a longer shelf life, so the development of chlamydospore agents has become a trend in fungal biocontrol agent research. Our findings demonstrate that *AfSwi6* can affect the chlamydospore formation and pathogenicity of *A. flagrans*, which provides a theoretical basis for the study of its biocontrol agents. Further, in-depth research on the mechanism of chlamydospore formation is crucial.

## 5. Conclusions

In this study, we characterized *AfSwi6* with homologous recombinant knockout, transcriptome sequencing, and multi-phenotype analysis. Our findings indicate that *AfSwi6* is a significant contributor to mycelium growth, stress response, chlamydospore formation, and pathogenicity. Meanwhile, the phenotypes of the *AfSwi6^C^* strains were consistent with those of the WT, indicating that the phenotypic changes caused by the deletion of the *AfSwi6* gene were restored after *AfSwi6* gene supplementation. Our results provide insights into SWI6 signaling regulation in *A. flagrans* and its mechanism of chlamydospore production.

## Data Availability

The raw data supporting the conclusions of this article will be made available by the authors on request.

## Supplementary Materials

The following supporting information can be downloaded at https://www.mdpi.com/article/10.3390/microorganisms12091765/s1.
