# Supplementary material for: AfSwi6 Regulates the Stress Response, Chlamydospore Production, and Pathogenicity in the Nematode-Trapping Fungus Arthrobotrys flagrans
Source: Microorganisms. 2024 Aug 26;12(9):1765. doi: 10.3390/microorganisms12091765 (PMC11433780; doi:10.3390/microorganisms12091765)
Supplement: Supplementary file 1 [file microorganisms-12-01765-s001.zip › microorganisms-3179121-supplementary.pdf]

Supplementary materials for

AfSwi6 regulates the stress response, chlamydospore production, and pathogenicity in the nematode-trapping fungus *Arthrobotrys flagrans*

Shao-Xiang Linghu, Yu Zhang, Jia-Fang Zuo, Ming-He Mo* and Guo-Hong Li*

State key Laboratory for Conservation and Utilization of Bio-Resources in Yunnan, School of Life Sciences, Yunnan University, Kunming, Yunnan 650091, China

*Correspondence: ligh@ynu.edu.cn (G.L.); minghemo@163.com (M.M.)

Content

1. Figure S1-S4.

2. Table S1-S15.

3. Materials and Methods.

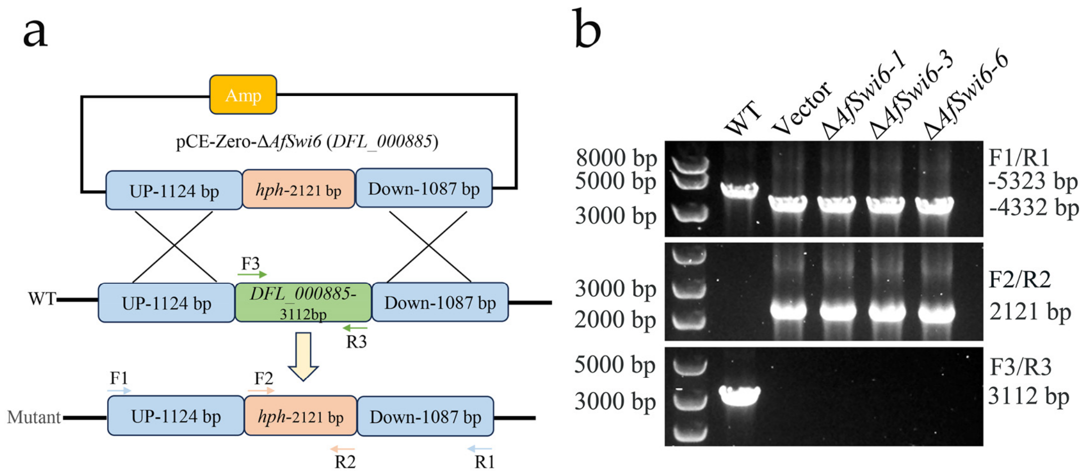

Figure S1. Disruption of *AfSwi6* gene in *A. flagrans*. (**a**) Diagram of the knockout pattern of the *AfSwi6* gene. (**b**) Detection of the knockout results of *AfSwi6* gene.

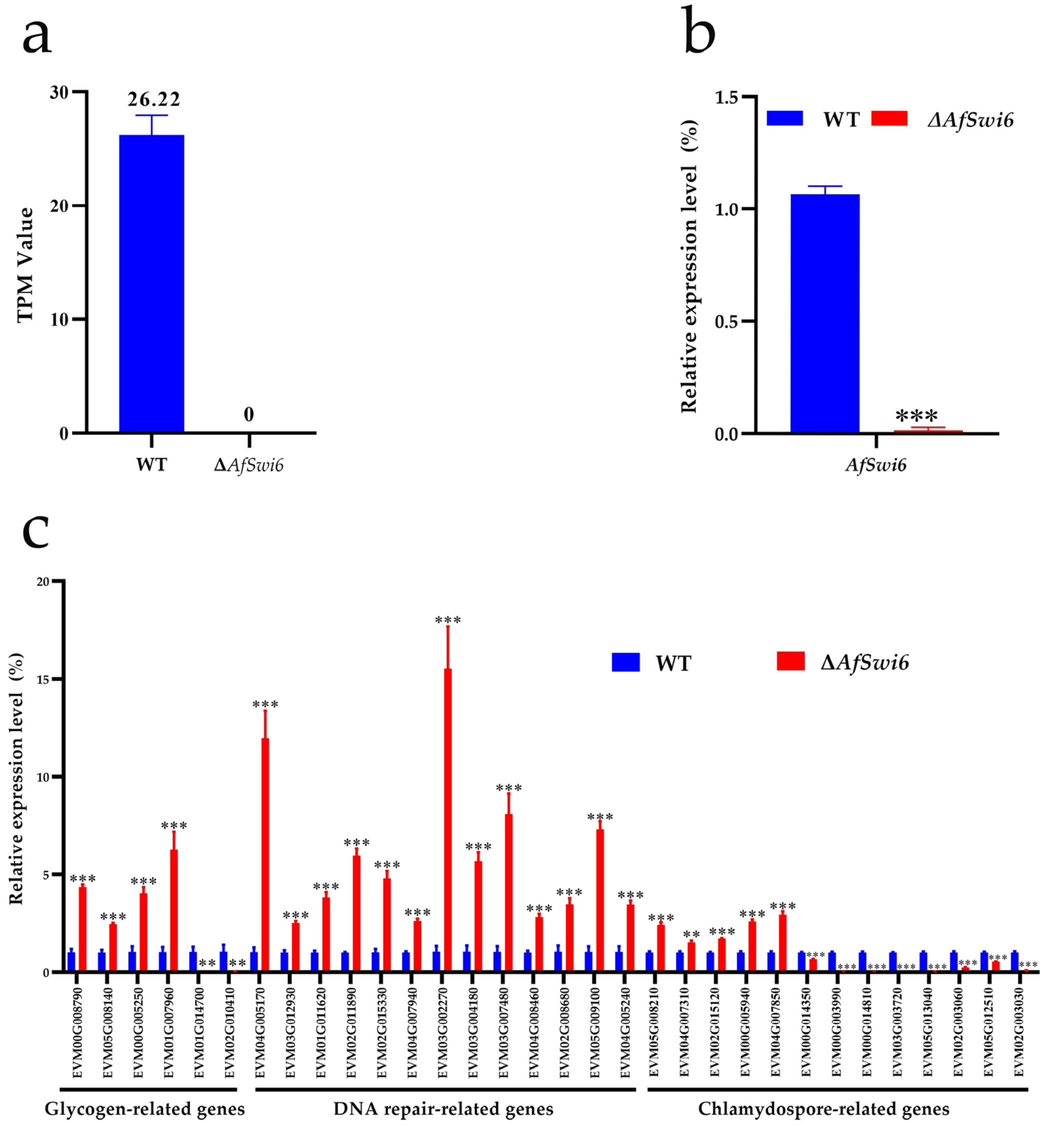

Figure S2. (**a**) Transcriptome data were used to confirm the expression level of *AfSwi6* gene in WT and Δ*AfSwi6* strains. (**b**) Detection of the relative expression levels of *AfSwi6* gene in Δ*AfSwi6* strains by qPCR. (**c**) Detection of the relative expression levels of glycogen-related genes, DNA repair-related genes and chlamydospore-related genes in Δ*AfSwi6* strains by qPCR (**, $P < 0.01$; * * *, $P < 0.001$).

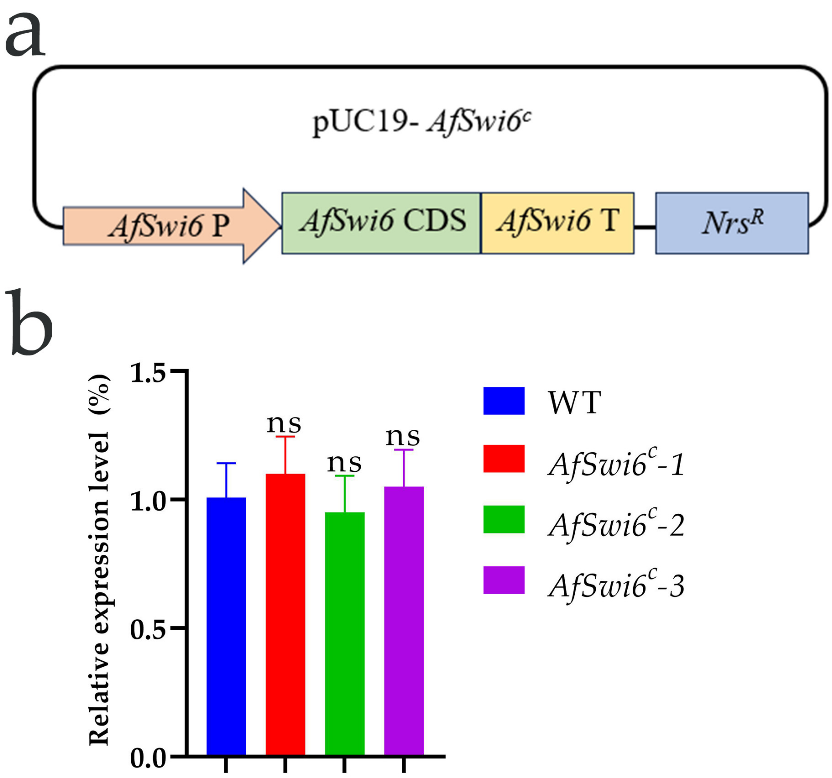

Figure S3. (**a**) Diagram of the complementation pattern of the *AfSwi6* gene in the *ΔAfSwi6* strain. (**b**) Detection of the relative expression levels of *AfSwi6* gene in *AfSwi6*^{C} strains by qPCR.

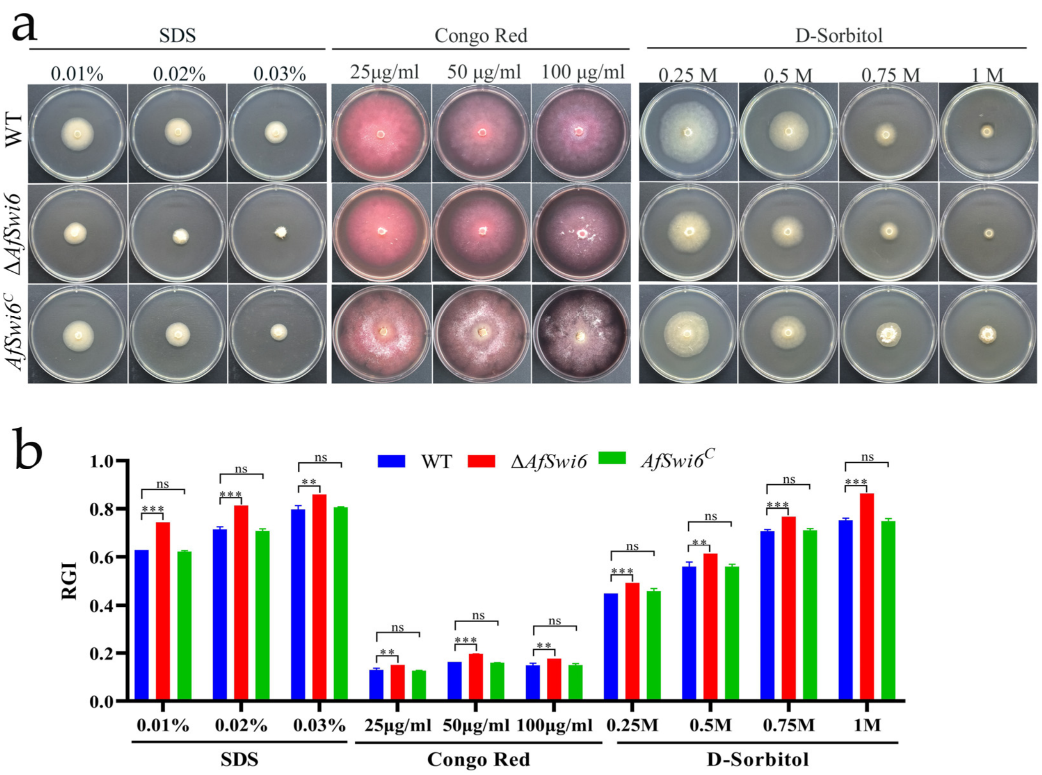

Figure S4. (**a**) WT, Δ*AfSwi6* and *AfSwi6*^{C} strains were cultured on media containing SDS, Congo red, and sorbitol at 28℃ for 5 days. (**b**) RGI comparison of WT, Δ*AfSwi6* and *AfSwi6*^{C} strains on different stress agents.

Table S1. The media and materials used in the experiment.

Name	Formula
PDA	Potato Dextrose Agar: Potato 200 g/L, glucose 20 g/L, agar 15-20 g/L
NGM	Nematode Growth Agar Medium: Peptone 2.5 g/L, NaCl 3.0 g/L, $CaCl_2$ 0.111 g/L, $MgSO_4$ 0.12 g/L, Cholesterol 0.005 g/L, KH_2PO_4 3.4 g/L, Agar 17.0 g/L
TYGA	Tryptone Yeast extract Glucose Agar: 1% Tryptone, 0.5% Yeast extract, 1% Glucose, 0.5% Molasses, 1.5% Agar
LMZ	Extracellular Enzyme Induction Medium: Gelatin, 20 g/L; Peptone, 8 g/L; Yeast extract, 1 g/L; $(NH_4)_2SO_4$, 0.5 g/L; $MgSO_4$, 0.5 g/L; $FeSO_4$, 0.01 g/L
PDB	Potato Dextrose Broth: Potato 200 g/L, glucose 20 g/L
CMA	Corn Meal Agar: Corn flour 20-30 g/L, Agar 15-20 g/L
WA	Water agar: Agar 10-20 g/L
STC buffer	1 M sorbitol, 50 mM $CaCl_2$, 10 mM Tris-HCl
PTC	10 mM Tris-HCl [pH 7.5], 50 mM $CaCl_2$, 50% [wt/vol] polyethylene glycol [PEG] 3350

Table S2. PCR primer sets used in this study.

Target gene	Sequence ID	Primers	Primer sequences (5'-3')	Uses
AfSwi6	DFL_000885(EVM05G009000)	KO0885-up-for	ttcggatcttccagaAAACAAGAAAGCAAGGAACAAAGG	Amplify the 5′ flank of *AfSwi6* gene
		KO0885-up-rev	tcttctgCAAGAATGCGACCTGTAAGGC	
		KO0885-hyg-for	ggtcgcattcttgCAGAAGATGATATTGAAGGAGCATTT	Amplify the *hph* cassette
		KO0885-hyg-rev	catcggtcatcgAAAGAAGGATTACCTCTAAACAAGTGTACC	
		KO0885-down-for	ccttctttCGATGACCGATGAATCCCAA	Amplify the 3′ flank of *AfSwi6* gene
		KO0885-down-rev	caactgccgttcgacGCCCGTGAATAGAAATCCCC	
		AfSwi6-F	GACCGACCCCAGATTCAGA	Amplify the *AfSwi6 gene*
		AfSwi6-R	AAGGAATGTTCGCCAACG	
Glycogen-related genes	EVM00G008790	8790-F	GGCATTCGCTTCGGTTGT	Amplify the glycogen-related genes
		8790-R	TGCTAAACGCTCGTGGGTC	
	EVM05G008140	8140-F	GTCTCGCCGCTTGTTTCT	
		8140-R	CCGTCGTCTTCGGTGTAA	
	EVM00G005250	5250-F	CTTTGCTGCTTCTAGCCG	
		5250-R	TCGTGCCTTCCTTTACCC	
	EVM01G007960	7960-F	CGGTTGTTTTCGGTTATTTG	
		7960-R	TCGATCCTTCGGTGTTTG	
	EVM01G014700	4700-F	ACCAACTTAGAAGAAGCCGACC	
		4700-R	CGCCAGGAGTATCAACAGTGC	
	EVM02G010410	0410-F	GCAGATCAACTATGGGGACC	
		0410-R	GCCGCAAAATCGAAGTCA	
DNA repair-related genes	EVM04G005170	5170-F	CCCCGTGGATCTCCAAAA	Amplify the DNA repair-related genes
		5170-R	TGCCGGAACCTCGTAACA	
	EVM03G012930	2930-F	GTCTTGTGCGGCTTCTGCT	
		2930-R	ACTGGCAACGCTGGTCTGA	
	EVM01G011620	1620-F	TCTGCGAAACTGGAACCT	
		1620-R	TGGATAATAGGGCGGCAT	
	EVM02G011890	1890-F	CCTTTCCAAAATGGCAGAT	
		1890-R	ACTTCCCGCAAACCCTAC	
	EVM02G015330	5330-F	ACCATGTGCGGGGAGTTT	
		5330-R	TCCATGCTTCGCCCTGTA	
	EVM04G007940	7940-F	ACCGCCGAAACAAAACAT	
		7940-R	TAGGACGCTGGGTGGAAT	
	EVM03G002270	2270-F	GTTATGTTGTGGTGGGGAGC	
		2270-R	ACCATGTGCGGGGAGTTT	
	EVM03G004180	4180-F	GAGCAGTCGGAAAAGGTG	
		4180-R	GGTCGTTCTTGTCGGGTT	
	EVM03G007480	7480-F	CCGACAAGCGGTTGGAAT	
		7480-R	TCCGTGAACCCGGACATA	
	EVM04G008460	8460-F	GACTTTGCCCACCGAGAA	
		8460-R	ACCGCGAGGGGTTTAACT	
	EVM02G008680	8680-F	CTGGGCTTTGGGTGTTGT	
		8680-R	GTGCCTCTGAATCCTTTATGG	
	EVM05G009100	9100-F	GCCACCCCACATTGAAAA	
		9100-R	ACCTTGGACGGACTACGCT	
	EVM04G005240	5240-F	ACAGTATTGCCTGGTGCG	
		5240-R	CGTTGTTTGTCAGATTTCGTAG	
Chlamydospore-related genes	EVM05G008210	8210-F	CCTCCGTAACCTTCCCATC	Amplify the chlamydospore-related genes
		8210-R	GCAGAGCAAACGAGTCCA	
	EVM04G007310	7310-F	CCACGCACCCCAATAACA	
		7310-R	GCCGAAGAAACGGAGGAT	
	EVM02G015120	5120-F	CCCCGTGGATCTCCAAAA	
		5120-R	TGCCGGAACCTCGTAACA	
	EVM00G005940	5940-F	ATGTTTGCTACACCCCATCA	
		5940-R	TGATGGGGTGTAGCAAACAT	
	EVM04G007850	7850-F	CGGTGGTGGTGTCGGTAAA	
		7850-R	CGTCGATAACGCATTGCT	
	EVM00G014350	4350-F	CGTCTCGTTCTATGGTGCCT	
		4350-R	GCTTGATGTCTCGGTGCA	
	EVM00G003990	3990-F	GACCCGAATGACCCTACCA	
		3990-R	GGGCATCCATCTCCAACA	
	EVM00G014810	4810-F	GGGTGCTTTCTCCCGCTTAT	
		4810-R	TGCAATTCCGCTTTGTGA	
	EVM03G003720	3720-F	GGAAGTGAACATTATTGGCTAC	
		3720-R	CTGAATCGGCAAGTCTCG	
	EVM05G013040	3040-F	GCCGTGATTGAGGTTTTG	
		3040-R	ATTCCCGTATCCGCCAGT	
	EVM02G003060	3060-F	CCAAGGGAACCTGCGATAC	

		3060-R	GAGGGAGGGGCTTTTAGA	
	EVM05G012510	2510-F	CACCATCTCCCCTGTCGTT	
		2510-R	TGGCCTGTGCTCATTTGC	
	EVM02G003030	3030-F	TCTCCCGCCGAAATGAAC	
		3030-R	CGCCATATCTATCGACCAAATT	
Complementation of *AfSwi6* gene	Promoter	RC-P-for	ttcggatcttccagaTTCAAGCTTCCGAAAAGTTATTTTG	Amplify the 5′ flank (2 kb) of *AfSwi6* gene
		RC-P-rev	gttgccatCAAGAATGCGACCTGTAAGGC	
	CDS (DFL_000885, EVM05G009000)	RC-CDS-for	gtcgcattcttgATGGCAACAACTGCTAGGCC	Amplify the CDS fragment of *AfSwi6* gene
		RC- CDS-rev	aaacggtgaaCTAGACCGAGGTCTGCGTCTCC	
	Terminator	RC-T-for	ctcggtctagTTCACCGTTTTTGCTACAACCG	Amplify the 3′ flank (1 kb) of *AfSwi6* gene
		RC-T-rev	tattctgggcctccatgtcTCCCCATCAGGCTCCATTC	
	NrsR	RC-Nrs^R-for	aGACATGGAGGCCCAGAATACC	Amplify the Nrs^R cassette
		RC-Nrs^R-rev	caactgccgttcgacCAGTATAGCGACCAGCATTCACA	

Table S3. The expression level of *AfSwi6* gene (Expression index: TPM).

Time of nematode induction	WT	Δ*AfSwi6*
0h	27.46	0
0h	26.93	0
0h	24.27	0
12h	32.59	0
12h	33.1	0
12h	31.98	0
24h	16.77	0
24h	23.08	0
24h	22.12	0

Table S4. The clean data of samples.

Sample	Total reads	Total mapped	Multiple mapped
WT_0 h_1	45579830	40667858(89.22%)	406444(0.89%)
WT_0 h_2	47470700	41610314(87.65%)	419406(0.88%)
WT_0 h_3	47477652	39998014(84.25%)	463104(0.98%)
WT_12 h_1	48645198	36680175(75.4%)	409697(0.84%)
WT_12 h_2	48883664	37228024(76.16%)	535647(1.1%)
WT_12 h_3	47707556	38483639(80.67%)	495924(1.04%)
WT_24 h_1	43846124	36040889(82.2%)	435868(0.99%)
WT_24 h_2	44138584	38837549(87.99%)	423405(0.96%)
WT_24 h_3	41178102	36112917(87.7%)	438494(1.06%)
Δ*AfSwi6*_0 h_1	43815824	40834246(93.2%)	423368(0.97%)
Δ*AfSwi6*_0 h_2	44455326	39213310(88.21%)	459681(1.03%)
Δ*AfSwi6*_0 h_3	52551882	48154595(91.63%)	628285(1.2%)
Δ*AfSwi6*_12 h_1	52200422	46048749(88.22%)	571941(1.1%)
Δ*AfSwi6*_12 h_2	50934610	45561661(89.45%)	544002(1.07%)
Δ*AfSwi6*_12 h_3	50776380	43908350(86.47%)	517214(1.02%)
Δ*AfSwi6*_24 h_1	48516076	45536425(93.86%)	639823(1.32%)
Δ*AfSwi6*_24 h_2	45552972	43014128(94.43%)	546368(1.2%)
Δ*AfSwi6*_24 h_3	43636136	41011470(93.99%)	516930(1.18%)

Table S5. The raw data and post-QC data of each sample are displayed.

Samples	Raw reads	Raw bases	Clean reads	Clean bases	Error rate(%)	Q20(%)	Q30(%)	GC content(%)
WT_0 h_1	46026672	6.95E+09	45579830	6.74E+09	0.0126	98.33	94.99	48.62
WT_0 h_2	47965418	7.24E+09	47470700	7.03E+09	0.0126	98.37	95.1	48.53
WT_0 h_3	47872536	7.23E+09	47477652	7.02E+09	0.0126	98.38	95.09	48.45
WT_12 h_1	49054834	7.41E+09	48645198	7.24E+09	0.0126	98.33	94.95	48.03
WT_12 h_2	49311148	7.45E+09	48883664	7.26E+09	0.0127	98.31	94.92	47.99
WT_12 h_3	48144826	7.27E+09	47707556	7.09E+09	0.0126	98.34	94.99	48.32
WT_24 h_1	44223668	6.68E+09	43846124	6.51E+09	0.0126	98.34	95.01	48.02
WT_24 h_2	44541692	6.73E+09	44138584	6.57E+09	0.0126	98.31	94.95	48.36
WT_24 h_3	41530568	6.27E+09	41178102	6.12E+09	0.0128	98.24	94.71	48.35
Δ*AfSwi6*_0 h_1	44217698	6.68E+09	43815824	6.51E+09	0.0127	98.31	94.92	48.77
Δ*AfSwi6*_0 h_2	44903604	6.78E+09	44455326	6.61E+09	0.0126	98.34	95.01	48.53
Δ*AfSwi6*_0 h_3	53032578	8.01E+09	52551882	7.8E+09	0.0126	98.34	95	48.86
Δ*AfSwi6*_12 h_1	52635912	7.95E+09	52200422	7.75E+09	0.0126	98.36	95.08	48.23
Δ*AfSwi6*_12 h_2	51379916	7.76E+09	50934610	7.57E+09	0.0126	98.31	94.93	48.45
Δ*AfSwi6*_12 h_3	51231060	7.74E+09	50776380	7.54E+09	0.0127	98.29	94.88	48.41
Δ*AfSwi6*_24 h_1	48965890	7.39E+09	48516076	7.2E+09	0.0126	98.32	94.96	48.58
Δ*AfSwi6*_24 h_2	45973678	6.94E+09	45552972	6.77E+09	0.0127	98.28	94.84	48.75
Δ*AfSwi6*_24 h_3	44012548	6.65E+09	43636136	6.5E+09	0.0127	98.28	94.82	48.66

Table S6. The DEGs of WT and Δ*AfSwi6*.

Groups	Total DEGs	Up	Down
WT_24 h_vs_WT_0 h	5688	2935	2753
Δ*AfSwi6*_24 h_vs_Δ*AfSwi6*_0 h	4743	2386	2357
WT_12 h_vs_WTh_0 h	5733	2941	2792
Δ*AfSwi6*_12 h_vs_Δ*AfSwi6*_0 h	4875	2559	2316

Table S7. The DEGs of WT and Δ*AfSwi6* at 12 h and 24 h.

Groups	Total DEGs	Up	Down
Δ*AfSwi6*_24 h_vs_WT_24 h	3310	1521	1789
Δ*AfSwi6*_12 h_vs_WT_12 h	3500	1886	1614

Table S8. GO analysis of the upregulated genes at 12 h (Δ*AfSwi6*_12 h_vs_WT_12 h) and 24 h (Δ*AfSwi6*_24 h_vs_WT_24 h) .

GO ID	Term type	Description	Ratio_in_study	Ratio_in_pop	Rich factor	Pvalue	Padjust
GO:0006260	BP	DNA replication	16/ 504	48/ 6553	0.333333	2.64E-07	0.000608
GO:0006284	BP	base-excision repair	7/ 504	9/ 6553	0.777778	4.81E-07	0.000608
GO:0051276	BP	chromosome organization	18/ 504	62/ 6553	0.290323	4.99E-07	0.000608
GO:0008094	MF	ATP-dependent activity, acting on DNA	22/ 504	60/ 6553	0.366667	2.89E-07	0.000608
GO:0140658	MF	ATP-dependent chromatin remodeler activity	11/ 504	24/ 6553	0.458333	4.97E-07	0.000608
GO:0051716	BP	cellular response to stimulus	44/ 504	221/ 6553	0.199095	8.95E-07	0.00071
GO:0006281	BP	DNA repair	40/ 504	143/ 6553	0.27972	9.45E-07	0.00071
GO:0006974	BP	cellular response to DNA damage stimulus	41/ 504	160/ 6553	0.25625	1.13E-06	0.00071
GO:0006310	BP	DNA recombination	14/ 504	42/ 6553	0.333333	1.50E-06	0.00071
GO:0006259	BP	DNA metabolic process	53/ 504	228/ 6553	0.232456	1.51E-06	0.00071
GO:0035861	CC	site of double-strand break	7/ 504	10/ 6553	0.7	1.50E-06	0.00071
GO:0090734	CC	site of DNA damage	7/ 504	10/ 6553	0.7	1.50E-06	0.00071
GO:0140097	MF	catalytic activity, acting on DNA	39/ 504	113/ 6553	0.345133	9.97E-07	0.00071
GO:0003677	MF	DNA binding	65/ 504	355/ 6553	0.183099	1.73E-06	0.000753
GO:0033554	BP	cellular response to stress	44/ 504	211/ 6553	0.208531	2.03E-06	0.000824
GO:0050896	BP	response to stimulus	45/ 504	262/ 6553	0.171756	2.32E-06	0.000866
GO:0006950	BP	response to stress	44/ 504	238/ 6553	0.184874	2.41E-06	0.000866
GO:0000781	CC	chromosome, telomeric region	11/ 504	29/ 6553	0.37931	4.82E-06	0.001547
GO:0003684	MF	damaged DNA binding	8/ 504	15/ 6553	0.533333	4.60E-06	0.001547
GO:0098687	CC	chromosomal region	12/ 504	37/ 6553	0.324324	1.17E-05	0.00357

Table S9. GO analysis of the downregulated genes at 12 h (Δ*AfSwi6*_12 h_vs_WT_12 h) and 24 h (Δ*AfSwi6*_24 h_vs_WT_24 h) .

GO ID	Term type	Description	Ratio_in_study	Ratio_in_pop	Rich factor	Pvalue	Padjust
GO:0032220	BP	plasma membrane fusion involved in cytogamy	3/ 384	3/ 6553	1	0.0002	0.199811
GO:0045026	BP	plasma membrane fusion	3/ 384	3/ 6553	1	0.0002	0.199811
GO:0003746	MF	translation elongation factor activity	5/ 384	17/ 6553	0.294118	0.002316	0.705689
GO:0006428	BP	isoleucyl-tRNA aminoacylation	2/ 384	2/ 6553	1	0.003425	0.745644
GO:0006044	BP	N-acetylglucosamine metabolic process	2/ 384	2/ 6553	1	0.003425	0.745644
GO:0034457	CC	Mpp10 complex	2/ 384	2/ 6553	1	0.003425	0.745644
GO:0004822	MF	isoleucine-tRNA ligase activity	2/ 384	2/ 6553	1	0.003425	0.745644
GO:1901071	BP	glucosamine-containing compound metabolic process	6/ 384	28/ 6553	0.214286	0.004873	0.818865
GO:0008135	MF	translation factor activity, RNA binding	11/ 384	76/ 6553	0.144737	0.004427	0.818865
GO:0005488	MF	binding	183/ 384	2666/6553	0.068642	0.004523	0.818865
GO:0090079	MF	translation regulator activity, nucleic acid binding	11/ 384	77/ 6553	0.142857	0.004902	0.818865
GO:0009116	BP	nucleoside metabolic process	12/ 384	89/ 6553	0.134831	0.005423	0.851051
GO:0043244	BP	regulation of protein-containing complex disassembly	3/ 384	7/ 6553	0.428571	0.005853	0.866464
GO:0007009	BP	plasma membrane organization	3/ 384	7/ 6553	0.428571	0.005853	0.866464
GO:0045182	MF	translation regulator activity	11/ 384	79/ 6553	0.139241	0.005971	0.866464
GO:0002181	BP	cytoplasmic translation	3/ 384	8/ 6553	0.375	0.008962	1
GO:0042759	BP	long-chain fatty acid biosynthetic process	2/ 384	3/ 6553	0.666667	0.009877	1
GO:0006449	BP	regulation of translational termination	2/ 384	3/ 6553	0.666667	0.009877	1
GO:1901657	BP	glycosyl compound metabolic process	12/ 384	91/ 6553	0.131868	0.010436	1
GO:0006040	BP	amino sugar metabolic process	6/ 384	34/ 6553	0.176471	0.012936	1

Table S10. GO analysis of the downregulated genes at 12 h (Δ*AfSwi6*_12 h_vs_WT_12 h) in Venn analysis.

GO ID	Term type	Description	Ratio_in_study	Ratio_in_pop	Rich factor	Pvalue	Padjust
GO:0006400	BP	tRNA modification	"19/703"	"36/6553"	0.527777778	2.42E-07	0.000490807
GO:0015935	CC	small ribosomal subunit	"12/703"	"20/6553"	0.6	1.19E-07	0.000490807
GO:0015934	CC	large ribosomal subunit	"18/703"	"36/6553"	0.5	2.42E-07	0.000490807
GO:0008033	BP	tRNA processing	"30/703"	"67/6553"	0.447761194	8.45E-07	0.000511072
GO:0009451	BP	RNA modification	"26/703"	"53/6553"	0.490566038	1.04E-06	0.000511072
GO:0006412	BP	translation	"70/703"	"157/6553"	0.445859873	1.16E-06	0.000511072
GO:0043604	BP	amide biosynthetic process	"73/703"	"174/6553"	0.41954023	1.19E-06	0.000511072
GO:0006518	BP	peptide metabolic process	"74/703"	"195/6553"	0.379487179	1.42E-06	0.000511072
GO:0034470	BP	ncRNA processing	"53/703"	"166/6553"	0.319277108	1.50E-06	0.000511072
GO:0043043	BP	peptide biosynthetic process	"72/703"	"160/6553"	0.45	1.64E-06	0.000511072
GO:0006396	BP	RNA processing	"73/703"	"295/6553"	0.247457627	1.64E-06	0.000511072
GO:0034645	BP	cellular macromolecule biosynthetic process	"73/703"	"179/6553"	0.407821229	1.81E-06	0.000511072
GO:0043603	BP	cellular amide metabolic process	"77/703"	"229/6553"	0.336244541	1.81E-06	0.000511072
GO:0006399	BP	tRNA metabolic process	"47/703"	"122/6553"	0.385245902	1.82E-06	0.000511072
GO:1901566	BP	organonitrogen compound biosynthetic process	"103703"	"395/6553"	0.260759494	2.04E-06	0.000511072
GO:0044260	BP	cellular macromolecule metabolic process	"120/703"	"563/6553"	0.213143872	2.15E-06	0.000511072
GO:0034660	BP	ncRNA metabolic process	"71/703"	"222/6553"	0.31981982	2.28E-06	0.000511072
GO:0044249	BP	cellular biosynthetic process	"139/703"	"732/6553"	0.18989071	2.39E-06	0.000511072
GO:0009059	BP	macromolecule biosynthetic process	"87/703"	"357/6553"	0.243697479	2.51E-06	0.000511072
GO:0044271	BP	cellular nitrogen compound biosynthetic process	"113/703"	"482/6553"	0.234439834	2.52E-06	0.000511072

Table S11. GO analysis of the downregulated genes at 24 h (Δ*AfSwi6*_24 h_vs_WT_24 h) in Venn analysis.

GO ID	Term type	Description	Ratio_in_study	Ratio_in_pop	Rich factor	Pvalue	Padjust
GO:0016072	BP	rRNA metabolic process	"32/857"	102/6553"	0.31372549	1.58E-06	0.002012775
GO:0006364	BP	rRNA processing	"31/857"	"96/6553"	0.322916667	2.04E-06	0.002012775
GO:0030687	CC	preribosome, large subunit precursor	"11/857"	"16/6553"	0.6875	4.21E-07	0.002012775
GO:0005730	CC	nucleolus	"35/857"	"91/6553"	0.384615385	6.91E-07	0.002012775
GO:0030684	CC	preribosome	"18/857"	"42/6553"	0.428571429	1.70E-06	0.002012775
GO:0006913	BP	nucleocytoplasmic transport	"17/857"	"45/6553"	0.377777778	2.50E-05	0.007838347
GO:0051169	BP	nuclear transport	"17/857"	"45/6553"	0.377777778	2.50E-05	0.007838347
GO:0000054	BP	ribosomal subunit export from nucleus	"9/857"	"15/6553"	0.6	2.57E-05	0.007838347
GO:0033750	BP	ribosome localization	"9/857"	"15/6553"	0.6	2.57E-05	0.007838347
GO:0022613	BP	ribonucleoprotein complex biogenesis	"15/857"	"37/6553"	0.405405405	2.82E-05	0.008178307
GO:0003700	MF	DNA-binding transcription factor activity	"43/857"	"184/6553"	0.233695652	0.000130922	0.028498948
GO:0044085	BP	cellular component biogenesis	"15/857"	"42/6553"	0.357142857	0.000157335	0.031965179
GO:0030246	MF	carbohydrate binding	"31/857"	"121/6553"	0.256198347	0.000176271	0.034657179
GO:0048017	BP	inositol lipid-mediated signaling	"5/857"	"6/6553"	0.833333333	0.00020258	0.038585138
GO:0030248	MF	cellulose binding	"25/857"	"91/6553"	0.274725275	0.000217998	0.040263659
GO:0030247	MF	polysaccharide binding	"25/857"	"92/6553"	0.27173913	0.000250098	0.044833829
GO:0000027	BP	ribosomal large subunit assembly	"7/857"	"12/6553"	0.583333333	0.000278239	0.048453407
GO:0031503	BP	protein-containing complex localization	"9/857"	"19/6553"	0.473684211	0.000290563	0.048531507
GO:0016491	MF	oxidoreductase activity	"109/857"	"605/6553"	0.180165289	0.000294613	0.048531507

Table S12. GO analysis of the common downregulated genes in Venn analysis.

GO ID	Term type	Description	Ratio_in_study	Ratio_in_pop	Rich factor	Pvalue	Padjust
GO:0032220	BP	plasma membrane fusion involved in cytogamy	3/ 384	3/ 6553	1	0.0002	0.199811
GO:0045026	BP	plasma membrane fusion	3/ 384	3/ 6553	1	0.0002	0.199811
GO:0003746	MF	translation elongation factor activity	5/ 384	17/ 6553	0.294118	0.002316	0.705689
GO:0006428	BP	isoleucyl-tRNA aminoacylation	2/ 384	2/ 6553	1	0.003425	0.745644
GO:0006044	BP	N-acetylglucosamine metabolic process	2/ 384	2/ 6553	1	0.003425	0.745644
GO:0034457	CC	Mpp10 complex	2/ 384	2/ 6553	1	0.003425	0.745644
GO:0004822	MF	isoleucine-tRNA ligase activity	2/ 384	2/ 6553	1	0.003425	0.745644
GO:1901071	BP	glucosamine-containing compound metabolic process	6/ 384	28/ 6553	0.214286	0.004873	0.818865
GO:0008135	MF	translation factor activity, RNA binding	11/ 384	76/ 6553	0.144737	0.004427	0.818865
GO:0005488	MF	binding	183/ 384	2666/ 6553	0.068642	0.004523	0.818865
GO:0090079	MF	translation regulator activity, nucleic acid binding	11/ 384	77/ 6553	0.142857	0.004902	0.818865
GO:0009116	BP	nucleoside metabolic process	12/ 384	89/ 6553	0.134831	0.005423	0.851051
GO:0043244	BP	regulation of protein-containing complex disassembly	3/ 384	7/ 6553	0.428571	0.005853	0.866464
GO:0007009	BP	plasma membrane organization	3/ 384	7/ 6553	0.428571	0.005853	0.866464
GO:0045182	MF	translation regulator activity	11/ 384	79/ 6553	0.139241	0.005971	0.866464
GO:0002181	BP	cytoplasmic translation	3/ 384	8/ 6553	0.375	0.008962	1
GO:0042759	BP	long-chain fatty acid biosynthetic process	2/ 384	3/ 6553	0.666667	0.009877	1
GO:0006449	BP	regulation of translational termination	2/ 384	3/ 6553	0.666667	0.009877	1
GO:1901657	BP	glycosyl compound metabolic process	12/ 384	91/ 6553	0.131868	0.010436	1
GO:0006040	BP	amino sugar metabolic process	6/ 384	34/ 6553	0.176471	0.012936	1

Table S13. GO analysis of the upregulated genes at 12 h (Δ*AfSwi6*_12 h_vs_WT_12 h) in Venn analysis.

GO ID	Term type	Description	Ratio_in_study	Ratio_in_pop	Rich factor	Pvalue	Padjust
GO:0016491	MF	oxidoreductase activity	114/ 780	605/ 6553	0.18843	2.48E-06	0.001115
GO:0005215	MF	transporter activity	83/ 780	408/ 6553	0.203431	2.61E-06	0.001115
GO:0022857	MF	transmembrane transporter activity	81/ 780	397/ 6553	0.20403	2.81E-06	0.001115
GO:0016021	CC	integral component of membrane	343/ 780	2203/ 6553	0.155697	4.84E-06	0.001123
GO:0031224	CC	intrinsic component of membrane	344/ 780	2213/ 6553	0.155445	4.97E-06	0.001123
GO:0006790	BP	sulfur compound metabolic process	27/ 780	94/ 6553	0.287234	1.37E-05	0.002693
GO:0004364	MF	glutathione transferase activity	5/ 780	5/ 6553	1	2.36E-05	0.004114
GO:0044282	BP	small molecule catabolic process	19/ 780	63/ 6553	0.301587	8.15E-05	0.011553
GO:0031204	BP	post-translational protein targeting to membrane, translocation	4/ 780	4/ 6553	1	0.000199	0.024304
GO:0042579	CC	microbody	9/ 780	20/ 6553	0.45	0.000227	0.027132
GO:0009092	BP	homoserine metabolic process	5/ 780	7/ 6553	0.714286	0.000403	0.043114
GO:0031231	CC	intrinsic component of peroxisomal membrane	5/ 780	7/ 6553	0.714286	0.000403	0.043114
GO:0005779	CC	integral component of peroxisomal membrane	5/ 780	7/ 6553	0.714286	0.000403	0.043114

Table S14. GO analysis of the upregulated genes at 24 h (Δ*AfSwi6*_24 h_vs_WT_24 h) in Venn analysis.

GO ID	Term type	Description	Ratio_in_study	Ratio_in_pop	Rich factor	Pvalue	Padjust
GO:0006974	BP	cellular response to DNA damage stimulus	36/ 588	160/ 6553	0.225	1.53E-06	0.00353
GO:0033554	BP	cellular response to stress	41/ 588	211/ 6553	0.194313	1.71E-06	0.00353
GO:0006259	BP	DNA metabolic process	44/ 588	228/ 6553	0.192982	2.40E-06	0.00353
GO:0051716	BP	cellular response to stimulus	42/ 588	221/ 6553	0.190045	3.15E-06	0.00353
GO:0140513	BP	nuclear protein-containing complex	76/ 588	323/ 6553	0.235294	2.02E-06	0.00353
GO:0032991	CC	protein-containing complex	125/ 588	885/ 6553	0.141243	3.47E-06	0.00353
GO:0006643	CC	membrane lipid metabolic process	13/ 588	38/ 6553	0.342105	1.41E-05	0.011846
GO:0006325	BP	chromatin organization	18/ 588	67/ 6553	0.268657	1.55E-05	0.011846
GO:0006950	BP	response to stress	41/ 588	238/ 6553	0.172269	3.89E-05	0.025975
GO:0000077	BP	DNA damage checkpoint signaling	8/ 588	17/ 6553	0.470588	4.69E-05	0.025975
GO:0042770	BP	signal transduction in response to DNA damage	8/ 588	17/ 6553	0.470588	4.69E-05	0.025975
GO:1903047	BP	mitotic cell cycle process	18/ 588	73/ 6553	0.246575	5.47E-05	0.026671
GO:0006497	BP	protein lipidation	10/ 588	27/ 6553	0.37037	6.46E-05	0.026671
GO:0006664	BP	glycolipid metabolic process	9/ 588	23/ 6553	0.391304	9.17E-05	0.026671
GO:0009247	BP	glycolipid biosynthetic process	9/ 588	23/ 6553	0.391304	9.17E-05	0.026671
GO:1903509	BP	liposaccharide metabolic process	9/ 588	23/ 6553	0.391304	9.17E-05	0.026671
GO:0006505	BP	GPI anchor metabolic process	9/ 588	23/ 6553	0.391304	9.17E-05	0.026671
GO:0006506	BP	GPI anchor biosynthetic process	9/ 588	23/ 6553	0.391304	9.17E-05	0.026671
GO:0046467	BP	membrane lipid biosynthetic process	10/ 588	28/ 6553	0.357143	9.25E-05	0.026671
GO:0006355	BP	regulation of DNA-templated transcription	27/ 588	137/ 6553	0.19708	9.86E-05	0.026671

Table S15. GO analysis of the common upregulated genes in Venn analysis.

GO ID	Term type	Description	Ratio_in_study	Ratio_in_pop	Rich factor	Pvalue	Padjust
GO:0006260	BP	DNA replication	16/ 504	48/ 6553	0.333333	2.64E-07	0.000608
GO:0006284	BP	base-excision repair	7/ 504	9/ 6553	0.777778	4.81E-07	0.000608
GO:0051276	BP	chromosome organization	18/ 504	62/ 6553	0.290323	4.99E-07	0.000608
GO:0008094	MF	ATP-dependent activity, acting on DNA	22/ 504	60/ 6553	0.366667	2.89E-07	0.000608
GO:0140658	MF	ATP-dependent chromatin remodeler activity	11/ 504	24/ 6553	0.458333	4.97E-07	0.000608
GO:0051716	BP	cellular response to stimulus	44/ 504	221/ 6553	0.199095	8.95E-07	0.00071
GO:0006281	BP	DNA repair	40/ 504	143/ 6553	0.27972	9.45E-07	0.00071
GO:0006974	BP	cellular response to DNA damage stimulus	41/ 504	160/ 6553	0.25625	1.13E-06	0.00071
GO:0006310	BP	DNA recombination	14/ 504	42/ 6553	0.333333	1.50E-06	0.00071
GO:0006259	BP	DNA metabolic process	53/ 504	228/ 6553	0.232456	1.51E-06	0.00071
GO:0035861	CC	site of double-strand break	7/ 504	10/ 6553	0.7	1.50E-06	0.00071
GO:0090734	CC	site of DNA damage	7/ 504	10/ 6553	0.7	1.50E-06	0.00071
GO:0140097	MF	catalytic activity, acting on DNA	39/ 504	113/ 6553	0.345133	9.97E-07	0.00071
GO:0003677	MF	DNA binding	65/ 504	355/ 6553	0.183099	1.73E-06	0.000753
GO:0033554	BP	cellular response to stress	44/ 504	211/ 6553	0.208531	2.03E-06	0.000824
GO:0050896	BP	response to stimulus	45/ 504	262/ 6553	0.171756	2.32E-06	0.000866
GO:0006950	BP	response to stress	44/ 504	238/ 6553	0.184874	2.41E-06	0.000866
GO:0000781	CC	chromosome, telomeric region	11/ 504	29/ 6553	0.37931	4.82E-06	0.001547
GO:0003684	MF	damaged DNA binding	8/ 504	15/ 6553	0.533333	4.60E-06	0.001547
GO:0098687	CC	chromosomal region	12/ 504	37/ 6553	0.324324	1.17E-05	0.00357

Materials and Methods

Plasmid Construction and Protoplast Transformation:

Knockout of the *AfSwi6* gene was performed using the primer groups Ko0885-up-for/rev, Ko0885-down-for/rev, and Ko0885-hyg-for/rev (Table S2) with PCR (Veriti™ Dx 96-well Thermal Cycler, 0.2 mL, Thermo Fisher Scientific) amplification. The three fragments act as upstream and downstream homologous arms, and hygromycin B resistance cassette (*hph*) respectively. The ClonExpress Ultra step-by-step cloning kit (Vazyme) was then used to assemble all the fragments into the pCE-Zero vector (digested with EcoRV). Subsequently, the knockout fragment was amplified by PCR with the primers ko0885-up-for and ko0885-down-rev and recovered at a concentration of 5 to 10 mg/mL (Figure S1a).

Hyphae Growth and Analysis of Resistance:

The PDA medium contained different stress agents including sorbitol (0.25, 0.5, 0.75, and 1 M), NaCl (0.1, 0.2, and 0.3 M), SDS (0.01%, 0.02%, and 0.03%), Congo red (25, 50, and 100 μg/mL), and H_2O_2 (2.5, 5, and 10 mM). The diameter of the colony was measured, and relative growth inhibition (RGI) was calculated. Each experiment was repeated three times and each treatment was also repeated at least three times.

Transcriptome Sequencing:

WT and Δ*AfSwi6* strains were grown in 200 mL PDB (Table S1) at 28°C, 180 rpm for 24 h. Then, 200 μL mycelia were coated onto the CMA medium (Table S1) plates (6 cm in diameter). The WT and mutant strains were sampled in three replicates for each time point. After culturing at 28 °C, for 48 h, about 1000 C. elegans were added. After the nematodes were added, mycelia were collected at 0, 12, and 24 h; samples were then frozen in liquid nitrogen and stored at -80°C. The mycelia were sequenced by Shanghai Majorbio Bio-pharm Technology Co., Ltd. (Shanghai, China) and the data were analyzed using the Majorbio platform (https:www.majorbio.com). Fungal mRNA sequencing is based on a high-throughput sequencing platform, and the process as follows: (1) Extraction of total RNA; (2) Oligo dT mRNA enrichment; (3) Fragmented mRNAs; (4) Reverse transcription synthesis of cDNA; (5) Linkage adaptor; (6) Fragment screening and library enrichment. The transcriptome sequencing of 18 samples was completed, and a total of 125.84 Gb Clean Data was obtained, and the Clean Data of all samples reached more than 6.12 Gb, and the percentage of Q30 bases was more than 94.71% (Table S4,5). The Clean Reads of each sample were compared with the specified reference genome, respectively, and the comparison rate ranged from 75.4% to 94.43%. A total of 9830 expressed genes were detected in this analysis. Based on the quantitative results of expression, differential gene analysis was performed between groups to obtain the genes that were differentially ex-pressed between the two groups. DESeq2 software was used to analyze the DEGs (differentially expressed genes) under conditions which included an upper/lower differential multiple of > 1.0 and an adjusted *P value* < 0.05.
